# Dependency of Glucose Homeostasis on Pancreatic Enzymes with Special Reference to Amylase; Study on Healthy and Exocrine Pancreatic Insufficient Pigs

**DOI:** 10.3390/biom16010172

**Published:** 2026-01-20

**Authors:** Piotr Wychowański, Stefan G. Pierzynowski, Kamil Zaworski, Robert Gallotto, Dominika Szkopek, Jarosław Woliński, Janine Donaldson, Tomasz Jacek, Kateryna Pierzynowska

**Affiliations:** 1 Department of Head and Neck and Sensory Organs, Division of Oral Surgery and Implantology, Institute of Clinical Dentistry, Gemelli Foundation for the University Policlinic, Catholic University of the “Sacred Heart”, 00168 Rome, Italy; 2 Department of Interventional Dentistry, Collegium Medicum, Nicolaus Copernicus University, 87-100 Bydgoszcz, Poland; 3 Department of Periodontology and Oral Diseases, Medical University of Warsaw, 02-091 Warsaw, Poland; 4 Department of Biology, Lund University, 22362 Lund, Sweden; stefan.pierzynowski@biol.lu.se; 5 Anara AB, 23132 Trelleborg, Sweden; 6 Department of Medical Biology, Institute of Rural Health, 20-090 Lublin, Poland; 7 Department of Animal Physiology, The Kielanowski Institute of Animal Physiology and Nutrition, Polish Academy of Sciences, 05-110 Jabłonna, Poland; k.zaworski@ifzz.pl; 8 Anagram Therapeutics, Inc., Natic, MA 01701, USA; rgallotto@anagramtx.com; 9 Large Animal Models Laboratory, The Kielanowski Institute of Animal Physiology and Nutrition, Polish Academy of Sciences, 05-110 Jabłonna, Poland; d.szkopek@ifzz.pl (D.S.); j.wolinski@ifzz.pl (J.W.); 10 Research Institute of Animal Husbandry, 111121 Balice, Poland; tomasz.jacek@iz.edu.pl; 11 Department of Physiology, School of Biomedical Sciences, Faculty of Health Sciences, University of the Witwatersrand, Johannesburg 2193, South Africa; janine.donaldson@wits.ac.za

**Keywords:** mixed meal tolerance test, MMTT, glucose metabolism, insulin release, amylase, pancreatic enzyme replacement therapy, PERT

## Abstract

We aimed to highlight the roles of the pancreatic enzymes, with special reference to amylase, on glucose homeostasis in healthy pigs and in pigs with exocrine pancreatic insufficiency (EPI). Healthy pigs fed a high-fat diet (HFD) were subjected to mixed meal tolerance tests (MMTTs) and pancreatic enzyme treatments, and then blood glucose and insulin concentrations were determined. Following the development of surgically induced EPI, the same experiment was then repeated on the pigs. A significantly lower net postprandial glycemic response was observed in pigs with EPI compared to healthy pigs. Net postprandial glycemic response was not affected by enzyme supplementation during the MMTTs in healthy pigs, but it was affected by adaptation to macronutrient components of the MMTT test meal, both in healthy and EPI pigs. Net postprandial glycemic response and insulin release curves reached higher levels in Creon-treated EPI pigs compared to amylase-treated EPI pigs. In summary, glucose homeostasis mechanisms in EPI pigs were downregulated compared to healthy animals. Creon supplementation during EPI significantly increased postprandial glucose level, while amylase treatment had the opposite effect, which could be explained by its metabolic actions.

## 1. Introduction

In clinical practice, different oral and parenteral nutritional strategies are currently used to estimate glucose homeostasis to introduce the correct medication or preventive treatment, as well as adequate nutritional support, as needed [1,2,3]. Glucose homeostasis is the continuous process that stabilizes fasting blood glucose to physiological levels between 80–100 mg/dL in most mammals, including humans, in response to changes in internal (expenditure) and external (absorption) conditions. Insulin and glucagon are commonly recognized as key regulators of glucose homeostasis [4,5].

Other factors that influence glucose homeostasis, such as the macronutrient content, kind of diet, and the adaptation to dietary macronutrients per se [6], are less well recognized. Digestive pancreatic enzymes with possible regulatory features are sometimes recognized as secondary players in glucose homeostasis, but are often neglected. However, it is generally accepted that specific diets constructed for patients are effective in promoting health [7,8,9]. Over the last few decades, there has been an explosion of dietary guidelines, recommendations, and formulas that are supposed to alleviate the main symptom (hyperglycaemia) of different congenital, metabolic, and/or age-related sicknesses, e.g., cystic fibrosis (CF), type 2 diabetes mellitus (DT2), metabolic syndrome, obesity, etc.

It is important to recognize that the nutritional effectiveness of various diets is highly dependent on the digestion and absorption of the macronutrient components. The qualitative and quantitative composition of pancreatic enzymes, as well as other enzymes of the gastrointestinal tract, including brush border enzymes, plays a primary role in the digestion of dietary macronutrients [10,11,12].

Pigs represent the accepted animal model for the study of human diseases and have been used for decades, e.g., due to their similarity to humans with regard to their immunology, anatomical size and structure, as well as their physiology [13,14]. The similarities between humans and pigs, specifically with regard to their gastrointestinal structure and function, resulting in comparable digesta transit times, nutrient absorptive processes, gut microbiota, etc., make the pig a more adequate, relevant animal model for humans, compared to the rat [14,15]. Recent studies performed on pigs highlighted the regulatory role of amylase in metabolism and its modulatory features, which are crucial for glucose homeostasis [16]. Amylase, administered orally, was shown to exhibit anti-incretin activity by reducing insulin secretion during an oral glucose tolerance test (OGTT) [16]. The results suggest that oral microbial amylase improves glucose homeostasis by reducing insulin resistance, and it (amylase) exhibits insulin-like features [16].

The benefit or rationality of amylase supplementation in people with exocrine pancreatic insufficiency (EPI) is often overlooked. Pancreatic amylase maintains physiological glucose utilization and, at the same time, reduces insulin release, which can protect pancreatic beta cells from exhaustion during long-lasting hyperinsulinemia [17]. Additionally, microbial amylase used in previous experiments reduced insulin resistance, thus protecting the host against the development of diabetes [18].

The main aim of the current study was to elucidate the specificity of amylase supplementation on glucose homeostasis as compared to Creon, a porcine-derived enzyme mixture commonly used in the treatment of EPI. We also attempted to emphasize the influence of test meal nutritional components on postprandial glycaemia and the importance of the use of the mixed meal tolerance test (MMTT) for the assessment of postprandial glycemic response, while simultaneously highlighting the modulatory action of pancreatic enzymes on the net postprandial glycemic response.

## 2. Materials and Methods

### 2.1. Animals

The present study was performed in strict accordance with the recommendations in the Guide for the Care and Use of Laboratory Animals of the National Institutes of Health and in accordance with ARRIVE guidelines. All efforts were made to minimize animal suffering. The study was approved by the Second Local Ethics Committee for Animal Experimentation in Warsaw, Poland (approval no. WAW2/025/2022, approval date 16 February 2022). The experiment was performed on 18 crossbred ((Polish Landrace × Yorkshire) × Hampshire)) pigs (*Sus scrofa domesticus*), purchased from a local herd (Karniewek, Poland), a mix of both genders (50%:50%, males were castrated on day 3 postpartum), aged eight weeks, and with a body weight of 15 ± 2.3 kg at the beginning of the study. The animals were randomly divided into 3 groups (Creon, amylase, and control groups); *n* = 6 pigs in each group. The experiments were performed on the pigs with their pancreas intact and then repeated on the same pigs after pancreatic duct ligation surgery and the development of EPI. The pigs were maintained on a 12 h day–12 h night cycle, with lights on from 06.00 to 18.00 h. The pigs were individually housed in pens (following surgery) for the whole duration of the experimental period. The individual pens were equipped with a feeding trough, drinking nipple, and constant heating lamp (150 W). The pigs were allowed to move freely within their pens and had visual and olfactory contact with each other.

Sample size was estimated using G∗Power software [19], version 3.1.9.4, for a one-way ANOVA at α = 0.05 with 95% power, assuming f (effect size) = 1.2, for three study groups. The calculation yielded the total number of animals as fourteen. However, we have increased the total number of animals up to 18 to keep the number sufficient for reaching the desired statistical power even in the case of animal loss.

### 2.2. Feed

**Figure 1:**
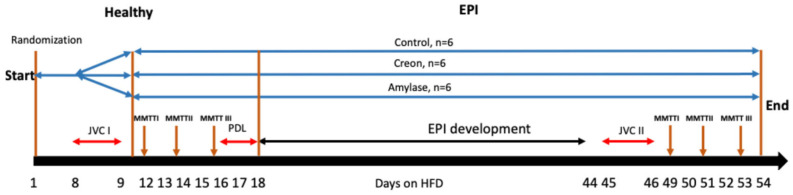
Experimental design and treatments. EPI—exocrine pancreatic insufficiency; JVC—jugular vein catheter implantation; MMTT—mixed meal tolerance test; PDL—pancreatic duct ligation.

During the study, pigs were adapted to a high-fat diet (HFD) (Kcynia, Morawski Plant, Poland, containing approximately 17.5% crude protein, 3.5% crude fiber, 20% crude fat, 50% starch, 4% ash and 5% water; the detailed composition is provided in Table 1) in an amount equivalent to 4% of their body weight daily, with 1% given at the morning meal (09:00–10:00 hr) and 3% at the afternoon meal (17:00–18:00). The HFD is often used to mimic the human diet, in terms of fat content, in metabolic studies using porcine models [20,21]. The feed was developed to fulfil the nutritional requirements of piglets with a body weight between 11 and 25 kg, according to the National Research Council requirements [22], with considerations for the experimental conditions and pig needs. Upon arrival at the experimental unit, the pigs were fed a cereal-based, pelleted, standard pig diet (Kcynia, Morawski Plant, Poland) and abruptly changed to an HFD on the first day of adaptation (Figure 1).

### 2.3. Selected Substrates for MMTT

The following substances were chosen to create the acute test meal used for the MMTTs to mimic a standard high-fat meal (Table 2): docosahexaenoic acid (DHA) and eicosapentaenoic acid (EPA)-Kinoko Life 2000 mg Omega-3 softgels; 500 mg EPA and 250 mg DHA per capsule (Kinoko Life, Malaga, Spain), Whey-California Gold Nutrition 100% Whey Protein Isolate, Unflavored (California Gold Nutrition, Irvine, CA, USA), Potato starch-Roots Circle Gluten-Free Potato Starch (Verno Goods LLC, Elizabeth, NJ, USA).

### 2.4. Enzymes

A microbial-derived alpha-amylase (Aspergillus oryzae, 4000 Units/dose, Amano Enzymes, Nishiki, Naka-Ku, Nagoya City, Aichi Prefecture, Japan) or porcine-derived enzyme mixture of Creon^®^ 25,000 (Abbot, Chicago, IL, USA; lipase (25,000 PhEur units), protease (1000 PhEur units) and amylase (18,000 PhEur units)) was administered to the pigs with the morning and evening meals, depending on the experimental design randomization. Pigs received a total of 100,000 lipase units/Creon^®^ dose during the study. Like pancreatic α-amylase, the microbial amylase studied hydrolyzes α-1,4-glucosidic linkages as the initial step in the hydrolysis of dietary starch, glycogen, and related polysaccharides. The control group (*n* = 6) was fed HFD alone; the Creon group (*n* = 6) was fed HFD + Creon^®^ (2 × 100,000 units daily); the amylase group was fed HFD + amylase (2 × 4000 units daily) at the morning and evening meals, respectively, for the entire duration of the experiment. A detailed study design is shown in Figure 1.

### 2.5. Blood Sampling

Blood samples were collected during the MMTT via a jugular vein catheter, at one hour and then again at one minute prior to feeding, and then at 5, 15, 30, 45, 60, and 120 min after feeding and transferred to BD Vacutainer^®^ glass Aprotinine K3EDTA tubes (BD Diagnostics, Franklin Lakes, NJ, USA). The blood samples were immediately placed on ice before they were centrifuged at 3000× *g* for 15 min at 4 °C, and plasma was separated and stored at −80 °C until further analysis.

Blood glucose concentrations were measured directly following blood sampling using a glucometer and test strips (Accu-Chek Aviva, Roche Diagnostics, Mannheim, Germany). Plasma insulin concentrations were measured using a porcine insulin ELISA kit (cat# 10-1200-01; Mercodia, Uppsala, Sweden).

### 2.6. Experimental Flow

From the first day, all pigs were randomised to either the control, Creon or amylase groups and adapted to the HFD. Additionally, animals from the Creon and amylase groups were adapted to their respective enzyme supplementation during morning and evening meals (Figure 1). All pigs underwent jugular vein catheter implantation (JVCI) [23] on days 8 and 9. Starting from day 12, and every second day thereafter, the respective MMTTs were performed on healthy pigs (Figure 1). The MMTTs were performed following an overnight fast and took place at the same time and in the same manner as the pigs’ normal morning meal. On days 17 and 18, all pigs were subjected to pancreatic duct ligation (PDL) surgery, as previously described [24]. After EPI development (defined as growth retardation, hyperphagia, and lowered immunoreactive trypsin in the blood, as measured 3–4 weeks after surgery), on days 45 and 46, complementary jugular vein catheters (JVC II) were implanted.

Starting from day 49, and every second day thereafter, identical MMTTs, with identical treatment and experimental order as those performed on the pigs in a healthy state, were then performed on the pigs in the EPI state.

### 2.7. Statistical Analysis

Statistical analysis was performed on the data generated from this study using an ordinary one-way ANOVA for normally distributed datasets or a Kruskal–Wallis test when data was not normally distributed. To report differences in medians between groups, a Kruskal–Wallis test was performed, followed by a pairwise Mann–Whitney U test. The actual differences between medians, as well as the Hodges–Lehmann difference is reported. The data distribution was assessed using the Shapiro–Wilk normality test. Outliers within data sets were identified using the ROUT method of regression (Q = 0.05%). A mixed effects analysis was conducted to examine the effects of the MMTT meal composition and enzymatic treatment on net postprandial glycemic response. All the analyses were carried out using GraphPad Prism 10.0, San Diego, USA. Data was not corrected for multiple comparisons as the study design included planning for the measurable outcomes [25]. Differences were considered significant if *p* ≤ 0.05; differences were considered as a trend when *p* ≤ 0.1 (0.05 ≤ *p* ≤ 0.10); data with a Gaussian distribution are expressed as mean ± standard deviation (SD); data with a non-Gaussian distribution are expressed as median ± interquartile range (IQR). Area under the curve (AUC) data is baseline adjusted.

## 3. Results

**Figure 2:**
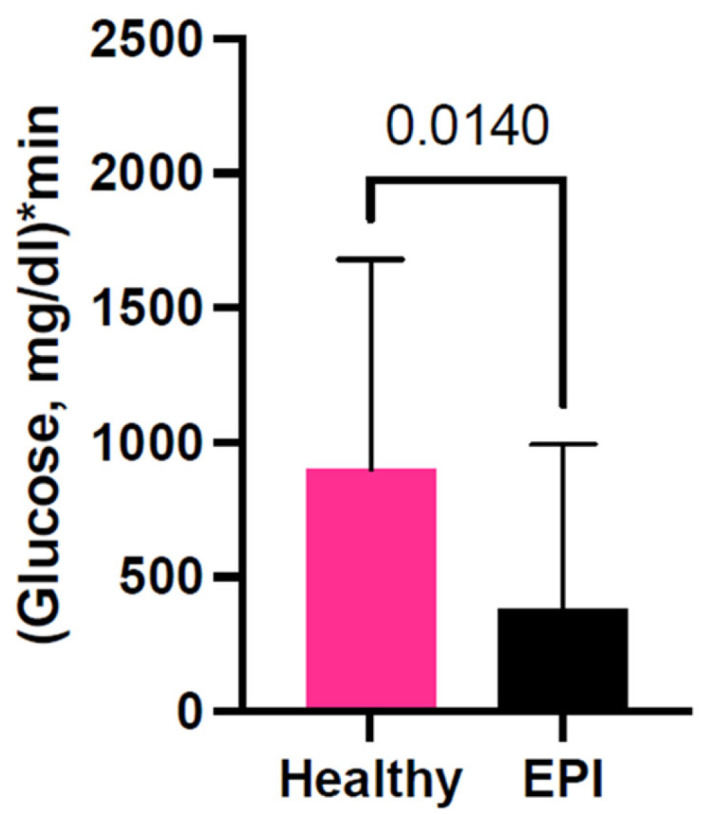
Net postprandial glycemic response AUCs during all MMTTs, with and without enzyme supplementation, 50 values per group. Area under the curve (AUC) data is baseline adjusted. Data are expressed as median ± interquartile range (IQR). Differences between the results were considered significant at *p* < 0.05. *p*-values are given with the result bars.

The median values of baseline-adjusted glucose AUCs describe the net absorption with basal metabolic values subtracted. AUC values are always directly proportional to absorption capacity and inversely proportional to expenditure/metabolic capacity during test time. AUCs obtained during different MMTTs are presented in Figure 2. Glucose AUC values obtained from healthy pigs were significantly higher when compared to those seen in the same pigs after EPI development. The median for postprandial glycemic AUCs was approximately 384 in EPI pigs and 900 in healthy pigs, with an actual median difference of −516.0, and a Hodges–Lehmann difference of −342.9 (*p* = 0.0140), independently of enzyme treatment or test meal composition.

**Figure 3:**
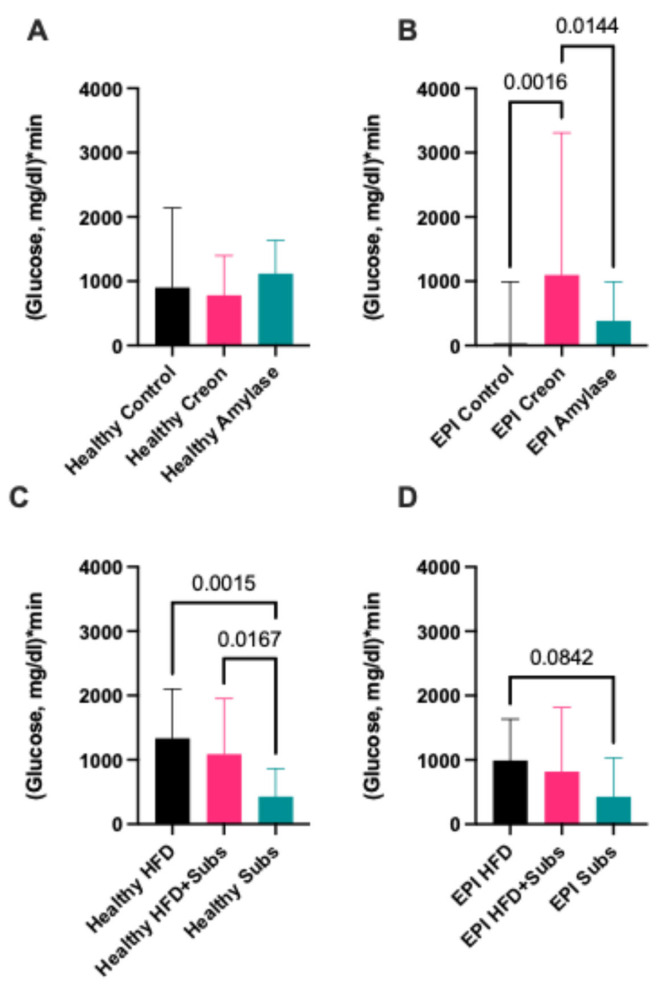
(**A**–**D**) Combined net postprandial glycemic response AUC values (14 values per result bar) obtained during MMTT in healthy and EPI pigs. (**A**) Summary of glucose AUCs describing the effects of enzymatic treatments in healthy animals on net postprandial glycemic response during all MMTTs; (**B**) summary of glucose AUCs describing the effects of enzymatic treatments in EPI animals on net postprandial glycemic response during all MMTTs; (**C**) summary of glucose AUCs describing the effects of different MMTTs composition in healthy animals on net postprandial glycemic response; (**D**) summary of glucose AUCs describing the effects of different MMTT compositions in EPI animals on net postprandial glycemic response. Data are expressed as median ± interquartile range (IQR). Differences between the results were considered significant at *p* < 0.05; *p* < 0.1 was considered a trend; *p*-values are given with the result bars.

The glucose AUCs in healthy pigs were not affected by Creon or microbial amylase supplementation (Figure 3A), but they were significantly affected by the type of macronutrients used during the various MMTTs (Figure 3C). The median postprandial glucose response in healthy pigs, during the MMTT with components to which the pigs were not adapted to at all (substrate (SUB)), was 427.5, while the median observed during the MMTT with the HFD (100% adaptation), was 1335.0, with an actual median difference of −907.5 and a Hodges–Lehmann difference of −967.5). The median observed during the MMTT with the HFD + SUB (60% adaptation) was 1089.0, with an actual median difference of −661.5 and a Hodges–Lehmann difference of −672.5 (Figure 3C).

In the same animals, after the development of EPI, during identical MMTTs, Creon treatment significantly increased the glucose AUC compared to that observed in control EPI pigs (actual median difference −1065, Hodges–Lehmann difference −992.8) and EPI pigs receiving amylase (actual median difference −713.0, Hodges–Lehmann difference −787.5,) (Figure 3B), possibly increasing absorption and minimising glucose expenditure compared to that observed in the control and microbial amylase groups.

There was a trend (*p* < 0.1) for reduced AUC in EPI pigs during the MMTT with the HFD + SUB compared to that observed during the MMTT with only the HFD (Figure 3D).

**Figure 4:**
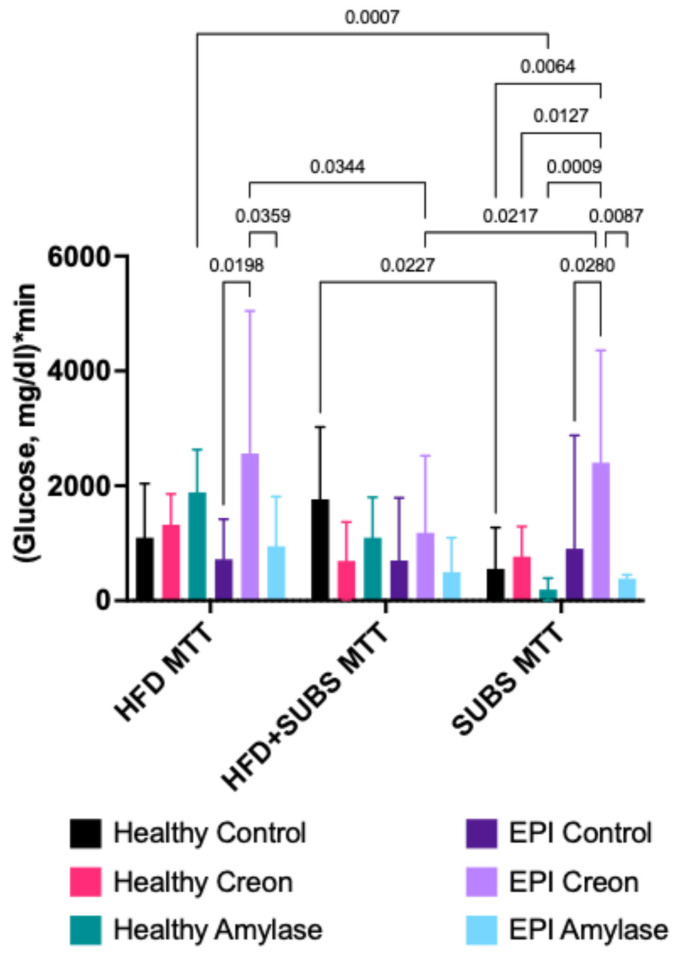
Net postprandial glycemic response AUCs describing the effects of MMTTs composition. Data are presented as mean ± standard deviation (SD) and are baseline adjusted. Differences between the results were considered significant at *p* < 0.05; *p* < 0.1 was considered a trend; *p*-values are given with the result bars.

A mixed effects analysis was conducted to examine the effects of MMTT meal composition and enzymatic treatment on net postprandial glycemic response. Results showed a significant interaction between MMTT composition and enzymatic treatment (F(10,38) = 2.1, *p* = 0.0449). Comparisons of simple effects on net postprandial glycemic response for specific MMTTs in healthy and EPI pigs are presented in Figure 4. The postprandial glycemic response of both healthy control pigs and healthy animals supplemented with amylase was significantly (*p* = 0.027 and 0.007, respectively) influenced by MMTT composition (Figure 4). At the same time, EPI control and EPI amylase animals demonstrated no MMTT composition-dependent changes in net postprandial glycemic response, while EPI Creon animals’ postprandial glucose curves were obviously different in all three types of MMTTs (*p* = 0.0344 and 0.0217, for HFD MTT vs. HFD + SUBS MMTT and HFD + SUBS MMTT vs. SUBS MMTT, respectively). EPI pigs receiving amylase, however, demonstrated significantly lower glucose response when compared to the Creon-supplemented group, in HFD and SUBS MMTT (Figure 4). It should be noted that due to the significant interactions between MMTT compositions and enzymatic treatments, no significant main effects were reported. No significant influence of random effect (animal) or its interactions with main effects (MMTTs composition and enzymatic treatment) was observed either.

**Figure 5:**
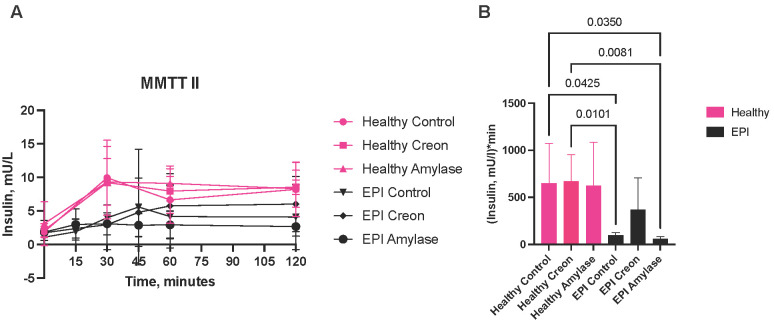
(**A**) Plasma insulin concentrations during mixed meal tolerance test (MMTT) with HFD + SUB; (**B**) comparison of area under the curve (AUC) for insulin release during mixed meal tolerance test (MMTT) with HFD + SUB. Data are expressed as mean ± standard deviation (SD). Area under the curve (AUC) data is baseline adjusted. Differences between the results were considered significant at *p* < 0.05; *p* < 0.1 was considered a trend; *p*-values are given with the result bars.

Amylase or Creon supplementation in healthy pigs did not affect insulin release (Figure 5A,B). Insulin release in EPI pigs during the HFD + SUB MMTT was lower (Figure 5A,B) than that observed in healthy pigs. Insulin secretion after Creon supplementation to EPI pigs was significantly higher compared to that observed in animals receiving amylase-supplemented feed or in control EPI pigs. The lowest insulin secretion in EPI pigs treated with microbial amylase corresponded to the lowest net postprandial glycemic response during the HFD + SUB MMTT.

## 4. Discussion

The global prevalence of diabetes mellitus and other metabolic diseases is rapidly growing. According to WHO recommendations, the oral glucose tolerance test (OGTT) is still considered to be a gold standard in the diagnosis of diabetes mellitus (https://www.who.int/news-room/fact-sheets/detail/diabetes (accessed on 9 January 2026)) [26]. However, it is well-known that the OGTT is not a perfect diagnostic tool, as it does not reflect the interactions between nutritional components and their metabolic path during digestion and absorption [27]. The OGTT results in the rapid absorption of glucose, followed by a fast insulin peak, and can cause reactive hypoglycaemia and gastrointestinal symptoms, such as bloating, nausea, and even anxiety in some patients [28,29]. Thus, many patients do not complete the initial OGTT or any follow-up investigations. The need for other measurements, allowing for the successful assessment of postprandial glucose response, is emerging.

To propose an alternative to the OGTT, attempts to introduce continuous glucose monitoring (CGM) [30,31,32] or even communication-inspired eye diagrams (sub-THz GlucoEye) [33] are ongoing. Efforts have also been made to standardize the MMTT and introduce it for use in clinical practice [27,30]. However, the test meal composition, nutritional value, adaptation to its components, and interactions between this meal and the health status of patients have not been analyzed to date.

In the present study, we tried to analyze the influence of MMTT meal composition and health status (enzymatic treatment) in the well-established porcine EPI model. We have demonstrated that postprandial glucose levels after the MMTT in healthy pigs were higher compared to those observed in EPI pigs and thus, must be decreased to physiological values of between 90 and 110 mg/dL for the maintenance of homeostasis (Figure 2). The net postprandial glycemic response curve in healthy pigs was not affected by exogenous enzyme supplementation (Figure 3A); however, it was significantly affected by meal composition and/or adaptation to the feed macronutrient content (Figure 3C).

Following the development of EPI, enzyme supplementation had different modulatory effects on the glucose homeostasis of the pigs. Creon, which is a mixture of many components derived from porcine pancreatic glands (containing enzymes and other biologically active components), significantly increased net postprandial glycemic response (possibly limiting host metabolism and lowering glucose tissue utilization) compared to microbial amylase (Figure 3B). The strongest homeostatic effect of microbial amylase on net postprandial glycemic response or the intensification of glucose expenditure was observed during the SUB MMTT (with unfamiliar macronutrients) in EPI pigs, when compared to the HFD MMTT or HFD + SUB MMTT, containing macronutrients to which the pigs’ gastrointestinal tract was already adapted for a long period of time.

In the last century, Mohan et al. [34] showed that after 6 months of Creon treatment, patients achieved better control of diabetes, improvement in abdominal symptoms, and overall sense of well-being. This included a significant reduction in post-prandial plasma glucose and glycosylated hemoglobin at six months vs. baseline [34]. In our study, Creon was found to increase net postprandial glycemic response during the HFD MMTT in EPI pigs compared to pigs treated with microbial amylase. The most controlled glucose AUCs were obtained in EPI pigs treated with microbial amylase, with the highest AUCs obtained in EPI pigs treated with Creon (Figure 4). This confirms that both exposure to MMTT test meal components and microbial amylase treatment interact to modulate postprandial blood glucose homeostasis. As previously mentioned, the separate effects of enzymatic treatment and MMTTs composition were found to be non-significant, but this could probably be explained by the significant interactions between these two factors, which, in turn, is an extremely important point for consideration in the possible clinical use of MMTTs. In the present study, Creon was shown to increase postprandial blood glucose levels and stimulate insulin release, which paradoxically suggests an enhanced insulin resistance. Amylase supplementation in EPI animals led to a decreased net postprandial glucose response, when compared to Creon, thus maintaining insulin sensitivity and preventing pancreatic β-cells from exhaustion. Creon contains active lipase, protease, and amylase, and the difference in effects observed for Creon and one of its main components may be explained by the presence of protease, which was shown to decrease insulin sensitivity [25]. Amylase, in turn, was shown to improve insulin sensitivity and glucose homeostasis [35,36,37].

Wortham & Sander [38] stated that “Insulin secretion must be tightly coupled to nutritional state to maintain blood glucose homeostasis. To this end, pancreatic β-cells sense and respond to changes in metabolic conditions, thereby anticipating insulin demands for a given physiological context.” Interestingly, in our study during the MMTT with the HFD + SUB, the lowest insulin levels corresponded to the lowest glucose AUCs in EPI pigs, during both the control experiment and the experiment with microbial amylase, whereas during the Creon experiment, the secretion of insulin tended to increase following the MMTT (Figure 5B). In healthy pigs, the long-term supplementation of neither Creon nor microbial amylase had any effect on insulin release and the corresponding net postprandial glycemic response. When the effects of the different substrates were compared in relation to microbial amylase vs. Creon supplementation, the results obtained confirmed the above statements.

MMTT meals, composed of new (different from those used for adaptation) types of macronutrients (SUB), definitively altered net postprandial glycemic response and the metabolic expenditure of glucose, downregulating glucose homeostasis. Thus, standardized MMTTs, with test meals composed of macronutrient components unfamiliar to subjects (patients), should be considered as highly relevant tests for obtaining comparable results concerning the precise estimation of glucose homeostasis regulation and insulin release, e.g., for pancreatic enzyme replacement therapy (PERT), metabolic syndrome DT2, etc.

The main limitation of the present study is the lack of opportunity to obtain a complete evaluation of the acute effects (non-adapted) of pancreatic enzyme secretion on glucose homeostasis. The lack of focus on the role of the other enzymes contained within Creon (e.g., lipase and protease) on insulin, incretins, and other gut hormones is another limitation that will be considered in our upcoming experiments. However, a good piece of work related to the above-mentioned limitations has been presented in the article by Pierzynowski et al., 2016 [39].

## 5. Conclusions

In summary, the pancreatic enzymes, with their possible regulatory features, are less well recognized as regulators of glucose homeostasis, which function in parallel to insulin and incretins, and their role is often neglected. The homeostatic regulation of glucose levels in EPI pigs maintained the net postprandial glycemic response at a low level (probably due to impaired digestive and/or absorption pathways), while glucose expenditure may even be increased compared to healthy animals. Creon supplementation in EPI pigs caused the attenuation of glucose homeostasis regulatory mechanisms, increasing the net postprandial glycemic response and probably leading to enhanced insulin resistance, which in turn, limits the metabolic expenditure of glucose. In contrast to Creon, microbial amylase-dependent regulatory mechanisms of glucose homeostasis maintenance decreased the net postprandial glycemic response and eventually increased the metabolic expenditure of glucose. Thus, microbial amylase exhibits both insulin-like and anti-incretin features.

## Figures and Tables

**Table 1:** Composition of the high-fat diet (HFD) (Kcynia, Morawski Plant, Poland).

**Energy and Chemical Composition (per 1 kg Feed)**	
Energy, MJ	16.8 (4012.7 kcal)
Water, g	50.0
Crude protein, g	175.0
Carbohydrates, g	500.0
Crude fat, g	200.0
Ashes, g	40.0
Crude fiber, g	35.0
Amino Acids (per 1 kg feed)	
Cysteine + Methionine, g	8.1
Threonine, g	8.7
Lysine, g	13.9
Leucine, g	14.2
Methionine, g	4.7
Total Nitrogen, g	38.8
Minerals (per 1 kg feed)	
Calcium, g	11.0
Chloride, g	3.2
Phosphorus, g	9.0
Potassium, g	3.1
Sodium, g	2.9
Magnesium, g	0.4
Iodine, mg	0.15
Iron, mg	100.0
Manganese, mg	3.5
Zinc, mg	85.0
Copper, mg	4.9
Selenium, mg	0.23
Vitamins (per 1 kg feed)	
Vitamin A, IU	2250
Vitamin D, IU	375
Vitamin E, IU	11
Vitamin K, mg	1.1
Biotin, mg	0.05
Niacin, mg	30.0
Pantothenic acid, mg	10.0
Riboflavin, mg	3.5
Thiamin, mg	1.0
Vitamin B_6_, mg	3.0
Vitamin B_12_, μg	15.5

**Table 2:** Composition of mixed meal tolerance tests (MMTTs) offered as morning meals, which accounted for 1% of the daily requirements of pigs weighing approximately 16 kg. The meals were isoenergetic. In between experiments, pigs were fed the HFD, 1% and 3%, respectively, for morning and evening meals.

	MMTT I HFD	MMTT II HFD + SUB	MMTT II SUB
High Fat Diet Components	28 g cereal protein+ 32 g fat–rapeseed oil+ 79 g starch cereals+ 6 g crude fiber+ 8 g ash+ 7 g water= 160 g	18 g cereal protein+ 20 g rapeseed oil+ 50 g starch cereals+ 3 g crude fiber+ 4 g ash+ 5 g water= 100 g	0
Substrate (SUB) Components	0	4 g DHA/EPA+ 10 g whey+ 32 g potato starch+ 8 g soya oil 3 g ash+ 3 g water= 60 g	4 g DHA/EPA+ 28 g whey+ 85 g potato starch+ 28 g soya oil 8 g ash+ 7 g water= 160 g

## Data Availability

The original contributions presented in this study are included in the article. Further inquiries can be directed to the corresponding authors.

## Abbreviations

The following abbreviations are used in this manuscript:

AUC	Area under the curve
CF	Cystic fibrosis
DT2	Diabetes type 2
EPI	Exocrine pancreatic insufficiency
HFD	High-fat diet
MMTT	Mixed meal tolerance test
OGTT	Oral glucose tolerance test
PDL	Pancreatic duct ligation
PERT	Pancreatic enzyme replacement therapy
SUB	Substrate
